# Systematic Analysis of Transcriptional and Post-transcriptional Regulation of Metabolism in Yeast

**DOI:** 10.1371/journal.pcbi.1005297

**Published:** 2017-01-10

**Authors:** Emanuel Gonçalves, Zrinka Raguz Nakic, Mattia Zampieri, Omar Wagih, David Ochoa, Uwe Sauer, Pedro Beltrao, Julio Saez-Rodriguez

**Affiliations:** 1 European Molecular Biology Laboratory, European Bioinformatics Institute (EMBL-EBI), Wellcome Genome Campus, Hinxton, Cambridge, United Kingdom; 2 Department of Biology, Institute of Molecular Systems Biology, ETH Zurich, Zurich, Switzerland; 3 RWTH Aachen University, Faculty of Medicine, Joint Research Center for Computational Biomedicine (JRC-COMBINE), Aachen; EMBL-Heidelberg, GERMANY

## Abstract

Cells react to extracellular perturbations with complex and intertwined responses. Systematic identification of the regulatory mechanisms that control these responses is still a challenge and requires tailored analyses integrating different types of molecular data. Here we acquired time-resolved metabolomics measurements in yeast under salt and pheromone stimulation and developed a machine learning approach to explore regulatory associations between metabolism and signal transduction. Existing phosphoproteomics measurements under the same conditions and kinase-substrate regulatory interactions were used to *in silico* estimate the enzymatic activity of signalling kinases. Our approach identified informative associations between kinases and metabolic enzymes capable of predicting metabolic changes. We extended our analysis to two studies containing transcriptomics, phosphoproteomics and metabolomics measurements across a comprehensive panel of kinases/phosphatases knockouts and time-resolved perturbations to the nitrogen metabolism. Changes in activity of transcription factors, kinases and phosphatases were estimated *in silico* and these were capable of building predictive models to infer the metabolic adaptations of previously unseen conditions across different dynamic experiments. Time-resolved experiments were significantly more informative than genetic perturbations to infer metabolic adaptation. This difference may be due to the indirect nature of the associations and of general cellular states that can hinder the identification of causal relationships. This work provides a novel genome-scale integrative analysis to propose putative transcriptional and post-translational regulatory mechanisms of metabolic processes.

## Author Summary

Phosphorylation is a broad regulatory mechanism with implications in nearly all processes of the cell. However, a global understanding of possible regulatory mechanisms remains elusive. In this study, we examined the potential regulatory role of kinases, phosphatases and transcription-factors in yeast metabolism across a variety of steady-state and dynamic conditions. The main novelty of our analysis was to infer putative regulatory interactions from *in silico* estimated activity of transcription-factors and kinases/phosphatases. This provided functional information about the proteins important for the experimental conditions at hand that had not been uncovered before. We showed that activity profiles are predictive features to estimate metabolite changes in dynamic experiments, while the same was not visible in steady-state conditions. We also showed that dynamic experiments could be used to recapitulate and provide novel TFs-metabolite and K/Ps-metabolite regulatory associations. We believe these findings illustrates the usefulness of this approach for future integrative studies interested in studying metabolic regulation.

## Introduction

Cells sense and react to extracellular *stimuli* with coordinated intracellular responses conveying transcriptional, protein and metabolic changes [1–3]. The continuous technological advances over the last few decades has contributed to the advent of the omics era with quantitative measurements of hundreds to thousands of transcripts, proteins and metabolites across a variety of steady-state and time resolved conditions [4–6]. The increasing accumulation of molecular measurements have provided unprecedented knowledge of the cellular molecular adaptation, nonetheless the robust identification of the regulatory interactions underpinning these changes is still a challenge [7,8]. Currently, the bottleneck has shifted from data acquisition to the development of statistically robust and computationally efficient mathematical approaches capable of providing an integrated analysis of the different types of biological data available.

Regulatory responses mediate the adaptation of many biological aspects of a cell, for example, metabolism may be regulated transcriptionally and post-transcriptionally. At present, most of the integrative analysis of metabolomics data-sets have focused on the role of transcriptional regulation [8–11]. Previous studies have focused on the regulatory implication of transcription-factors (TFs) to model the metabolic transition between different steady-state conditions [10]. Moreover, these regulatory interactions may occur in the inverse direction where metabolites directly impact the activity of global cellular regulators, such as TOR1 [11,12]. Nevertheless, transcript levels have been shown to poorly predict metabolic fluxes in the central carbon metabolism and that glycolytic enzymes are predominantly regulated at the post-transcriptional level [3,13,14]. Signal transduction by reversible protein phosphorylation is a key cellular regulatory mechanism and has been shown to modulate the glycolytic flux by regulating metabolic enzymes [7]. Recent studies have explored the implication of phosphosites in the enzymatic activity of kinases/phosphatases (K/Ps) by integrating with metabolomics measurements and *in silico* estimated metabolic fluxes [7,15]. Nonetheless, data acquisition and subsequent integrated analysis of phosphoproteomics data-sets are much sparser than transcriptomics and are still lagging behind [7,15].

**Fig 1:**
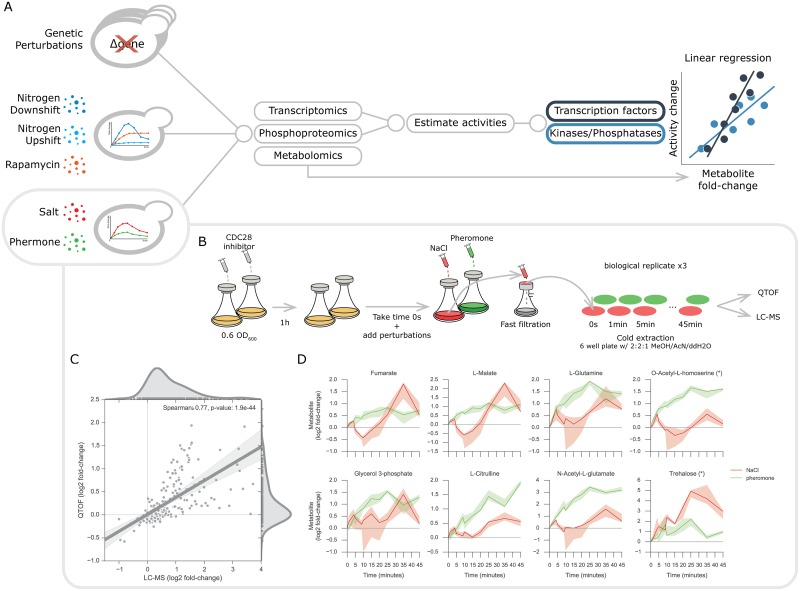
Analysis and experimental design and data consistency. (A) Representation of the different types data-sets used in the analysis. Transcriptomics and phosphoproteomics data-sets are used to estimate transcription factor and kinase/phosphatase activity changes, which are then separately associated with the respective metabolomics data-set using multilinear regression models. (B) Experimental design used to acquire the intracellular metabolomics measurements. CDC28 analog sensitive yeast strains inoculated in shake flasks were treated with the CDC28 inhibitor. The unperturbed initial time points were taken 1 hour after the CDC28 inhibitor and before adding the NaCl and pheromone. Sample filtration, metabolite extraction and MS injection were performed in parallel on the samples from independent triplicate experiments. (C) Representative metabolite profiles of untargeted metabolomics experiments. (D) Metabolites fold-changes correlation between targeted and untargeted metabolomics. Ions mapping to more than one metabolite are marked with an asterisk (*) and were not considered for any downstream analysis.

Transcriptional and translational regulatory interactions of metabolism can, in principle, be comprehensively explored using available high-throughput data-sets and methods [4,5,11]. However, current methods have yet to integrate gene-expression and phosphoproteomics measurement to infer regulatory interactions of metabolism. In this study, we set out to address this issue. We propose a computational approach to systematically identify putative post-transcriptional and post-translational regulatory mechanisms of metabolism (Fig 1A). To this end, we characterised the metabolomics adaptation of yeast under salt and pheromone conditions and further expanded it to consider a compendium of experimental data-sets [4–6,11,12], comprising a total of 143 unique conditions. Firstly, our computational approach predicted the *in vivo* activity of TFs and K/Ps. For that purpose, we considered prior-knowledge on regulatory interactions and mathematical approaches that have been developed to predict the activity status of transcription factors [16,17] and kinases [18,19] (Fig 1A). The activity of regulatory proteins is difficult to measure directly, yet provides functional information about the protein regulators involved in a cellular response. Subsequently, regulator activities were integrated with the metabolomics measurements using a machine learning approach to infer putative regulatory interactions. Our approach accurately estimates the activity status of known regulatory proteins, and identifies protein-metabolite associations capable of robustly estimating metabolic phenotypes of previously unseen conditions.

## Results

### Generation of dynamic metabolomics upon salt and pheromone perturbation and integration with a compendium of existing data-sets

To explore the functional implication of post-translational regulatory mechanisms in metabolism we set out to obtain paired phosphorylation, expression and metabolomics data under the same experimental conditions. For osmotic and pheromone conditions we resorted to existing dynamic phosphoproteomics measurements and we experimentally determined the intracellular metabolite changes. Both salt and pheromones are known to promote changes in phosphorylation of MAPK pathway and specifically share STE11, STE20 and CDC24 protein kinases [20,21]. The metabolic adaptations upon salt perturbation are arguably better characterised than pheromone and these involve the regulation ion membrane transporters and the production and retention of glycerol [21].

Wild-type strains of *S*. *cerevisiae* displays long periods to initiate the signalling response when stimulated with pheromone, while salt response is almost immediate [22,23]. To ensure that both responses began at comparable time-scales, yeast strains carrying a CDC28 analog sensitive version were used and CDC28 was inhibited with an ATP analog (Fig 1B). The dynamic response of metabolism was captured for both conditions pairing and expanding the time-points acquired in the phosphoproteomics data-set, i.e. 0 and 25 seconds and 1, 4, 5, 9, 10, 15, 20, 25, 35, and 45 minutes, 0 seconds represents the unperturbed state immediately before the stimuli are added. Cell material was extracted with fast-filtration and analysed with targeted (LC-MS/MS) [24] and untargeted (QTOF-MS) [25] mass-spectrometry (see Methods). Robustly identified ions spectra mass were then matched and annotated to an existing database [25]. In total, we measured with LC-MS/MS 54 metabolites and with QTOF-MS 11,190 ions for which 452 were mapped to metabolites using the genome-scale model iMM904 [26]. After robust quality assessment of the metabolite mass peaks and stringent identification of matching ion mass, we retained 26 metabolites for the downstream analysis from LC-MS/MS and 196 ions mapping to 74 metabolites from the QTOF-MS (see Methods). In order to estimate the reliability of the metabolite measurements, we compared the metabolic fold-changes measured in both targeted and untargeted MS (Fig 1C). A total of 11 unique metabolites were quantified with both methods and these showed strong concordance (spearman’s rho = 0.77, *p*-value < 1.9e-44). On the untargeted data-set, 33 ions were defined as significantly changing in at least one of the time-points analyzed (see Methods). These include several examples of metabolites known to be regulated under these conditions (Fig 1D). In general, there was a lack of measured products and reactants from the same reaction. However, fumarate and malate were reliably measured and both showed similar profiles [27]. Glycerol 3-phosphate displays an accumulation over time under salt stimulation, consistent with known signalling regulation of GPD1 leading to the production of glycerol [21,28,29]. Yeast cells also produce and accumulate trehalose under different types of stress conditions, including osmotic stress, and this is visible with the trehalose profile [21,30]. While the metabolic implications of the pheromone stimulation in yeast are generally poorly understood, the pheromone MAPK pathway is known to undergo regulation [31,32]. TOR and the pheromone MAPK signalling pathways have been shown to crosstalk [20]. Therefore, it is interesting to see that metabolites involved in the biosynthesis of amino-acids, such as, L-glutamine, N-acetyl-L-glutamate and L-citrulline significantly accumulate over time after pheromone stimulation. Some of these have been previously shown to directly influence TOR1 activity [12]. These results recapitulate previous findings and therefore support the usefulness of this metabolomics data-set to understand the metabolic adaptation to salt and pheromone.

To compare the responses between time-resolved experiments and steady-state genetic perturbations as well as to test the inference methods across different conditions, we expanded the analysis across a range of different cellular perturbations. Salt and pheromone data-sets were integrated with a compendium of biological experiments including time-resolved measurements related to nitrogen metabolism and steady-state genetic perturbations. To this end we considered a panel of 115 K/Ps knockouts, for which molecular changes at the transcript [5], phosphorylation [4] and metabolite [6] were characterised (Fig 1A) (see Methods) as well as metabolomics, transcriptomics and phosphoproteomics data-sets for three perturbations around nitrogen metabolism [11,12]. In these studies, yeast cells were perturbed by varying the growth medium from poor to rich nitrogen growing conditions (nitrogen upshift) and vice-versa (nitrogen downshift). Yeast cells were also stimulated with rapamycin, thereby inhibiting TOR1, a condition that resembles the nitrogen downshift (Fig 1A). Combining all the experimental data-sets together, we obtained a total of 143 different conditions for which metabolic, phosphorylation and gene expression measurements are available, except for salt and pheromone conditions where transcriptomics is not available (S1 Fig). These data-sets provide the basis for the systematic and comprehensive analysis of transcriptional and post-transcriptional regulatory interactions with metabolism.

### Inferring activity of transcription-factors, kinases and phosphatases

**Fig 2:**
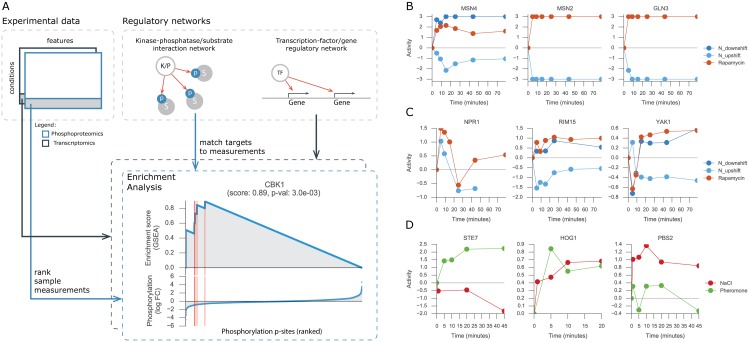
Protein activity analysis. (A) Representation of the workflow used to estimate protein activities using as input an experimental data-set and a regulatory network. Regulatory network will contain either the kinase-phosphatase/substrate interactions or transcription factor/gene associations. GSEA with random permutations is used for each protein in each condition. Red vertical lines represent the targets of the protein in the ascending sorted data-set. (B, C, D) Estimated activity profile of representative proteins for each experiment used in the analysis.

Changes in gene expression and in protein phosphorylation can be combined with metabolic measurements to identify possible regulatory associations. However, identification of functional regulatory interactions is hampered by the fact that expression is a poor proxy for TFs activity [16,17] and phosphorylation sites often display no functional impact in protein activity [7,33]. Therefore, to circumvent these limitations we have predicted the changes in activity of TFs and K/Ps. Enzymatic activity of K/Ps were estimated resorting to a comprehensive set of manually curated K/Ps-substrates interactions from PhosphoGrid [34]. TF activities were inferred using a regulatory network obtained by combining gene-expression data from TF knock-out experiments and TF binding sites from ChIP-chip experiments (see Methods). The changes in activity of a regulator can be estimated by considering the changes of its targets [16,18,35]. For example, by analysing the phosphorylation changes of reported target sites of a protein K/P, one can predict whether the K/P is changing significantly (Fig 2A).

Considering the reported targets of TFs and K/Ps we used the gene-set enrichment analysis (GSEA) [36] approach to quantify and estimate the significance of the activity of 91 TFs and 103 K/Ps across all conditions (see Methods). The phosphoproteomic data-sets contain 85.2%, 49.2% and 20.0% of missing values in the genetic, nitrogen and salt/pheromone perturbations, respectively. For this reason, the activity scores of K/Ps could not be predicted in 3,227 (48.0%), 498 (25.2%) and 434 (7.7%) cases for the genetic, nitrogen metabolism and salt/pheromone perturbations, respectively. We note however, that the estimated activities do not always rely on measuring the same set of reported targets, and hence the estimated activities matrices are less sparse than the original measurements. For the dynamic experiment of salt and pheromone stimuli there are no transcriptomics available, thus TFs activity could not be calculated.

Nitrogen downshift and rapamycin are similar conditions that inhibit TOR1 activity; in contrast, nitrogen upshift displays increased TOR1 activity. Thus, it is reassuring that the predicted protein activities tend to have similar changes in time for the nitrogen downshift and rapamycin condition, and opposite changes for the nitrogen upshift (Fig 2B and 2C). Several of the predicted activities are in line with known condition dependent activity changes. Examples include the TOR mediated inhibition of MSN2, MSN4 and GLN3 TFs [37] (Fig 2B) and the kinases NPR1 [38], RIM15 [39] and YAK1 [40] (Fig 2C). Moreover, HOG1 and PBS2, central kinases in the response to osmotic stress, display increased activity profiles [21,22] (Fig 2D). Similarly, the STE7 MAPK kinase of the pheromone pathway is predicted to be activated during pheromone stimulation (Fig 2D). These examples suggest that the TFs and K/Ps activities are well predicted and can be used to explore regulatory associations with metabolic changes. Regulator activities provide functional information that can be integrated with metabolic changes to infer functional regulatory interactions. Nevertheless, the activity of the regulators, as their expression and phosphorylation measurements, may be confounded by general cellular states (e.g. growth rate) and therefore lead to indirect associations. In the next section we tested the impact of growth rate on activity estimates and metabolic measurements.

### Growth rate implications in intracellular changes

General effects in the cell, such as cell cycle and growth rate, can act as confounding factors when searching for regulatory associations between TFs and K/Ps and metabolic changes. In particular, gene expression changes, which upon different perturbations have been shown to be tightly correlated with growth rate due to changes in the distribution of cells over the cell cycle phases [41,42]. Considering that relative growth rate measurements are available for the genetic perturbations experiments and for each time point of the dynamic nitrogen metabolism experiments, we set out to assess how much of the variation in the data-sets can be explained by growth rate alone. To this end, we performed Principal Component Analysis (PCA) on TF and K/P activities as well as metabolomics measurements. We then measured the correlation between relative growth rate and each of the top three principal components (PCs) (S2 Fig). Genetic perturbations metabolomics data-set PC 1 displayed a moderate correlation (Pearson’s r = 0.25, *p*-value < 7.0e-3) with the relative growth rates of the knockout strains. Growth rate displayed stronger correlations (Pearson’s r = 0.35 and -0.54, *p*-values < 1.2e-4 and 1.1e-4) with PC 1 of K/P and TF activities (S2 Fig). The same analysis was performed for the dynamic nitrogen metabolism data-set where metabolomics PC 2 displayed a strong correlation with the relative growth rate over time (Pearson’s r = 0.72, p-value < 8.0e-4). For the estimated K/P and TF activities, PC3 and PC2 showed also strong correlations with growth (Pearson’s r of 0.51 and 0.69, *p*-values < 3.2e-2 and 1.6e-3) (S2 Fig).

In summary, the variance in molecular measurements for the steady-state genetic perturbation experiments is more strongly influenced by the growth rate than the measurements performed in the dynamical perturbations. For the steady-state conditions, the gene-expression changes are the molecular changes, confounded mostly by the growth rate.

For the subsequent association analyses we tested the impact of removing the growth rate from each data-set to rule out any confounding effects it may have on the identification of direct functional interactions. To this end, we regressed-out growth rates from the original metabolite measurements and estimated TFs and K/Ps activities using linear regression models and growth as a covariate.

### Estimating metabolic changes from transcription-factors, kinases and phosphatases activities

**Fig 3:**
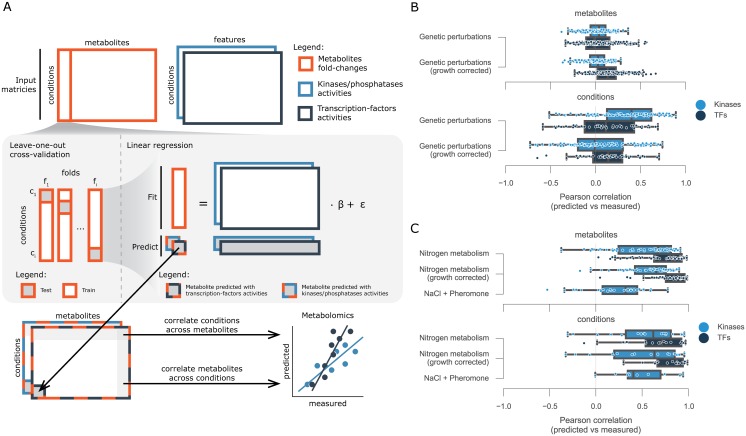
Computational framework for finding associations between protein and metabolites. (A) Diagram of the analysis. For each metabolite measured a multilinear regression analysis was performed using leave-one-out cross-validation. Changes in activity for kinases/phosphatases and transcription factors were used independently to estimate the metabolite fold-change. Independently predicted metabolite fold-change matrices were then correlated metabolite and condition wise with the measured values. Distributions of the correlation values between predicted and measured metabolite fold-changes for the (B) genetic perturbations and for the (C) time-resolved experiments.

Next we explored the correlations between TFs and K/Ps enzymatic activities and metabolic changes for each of the three experiments: genetic, nitrogen metabolism and salt/pheromone perturbations. To identify the relationships we used linear regression models that consider the estimated activities as features and metabolite fold-changes as observations (S1 Fig). Considering the low number of samples available, specifically for the time resolved experiments, a cross-validation procedure of leave-one-out (LOO) was used (see Methods). This allowed us to understand how much information can be transferred within each experiment to predict the metabolite variations in an independent testing sample. Thus, for each experiment and each metabolite, independent training and test data-sets were generated leaving one sample out at a time for test, i.e. single KO or time-point, and thereby generating a complete metabolomics matrix with estimated fold-change values (Fig 3A). The analysis was performed using TFs and K/Ps activities independently. In each experiment four different types of input matrices are used to predict each metabolite, i.e. K/P or TF activities with and without growth normalisation, with the exception of the dynamic experiment with NaCl and pheromone for which neither growth rate nor transcriptomics measurements were available. To minimize possible effects of over-fitting while training the linear models an Elastic Net feature regularisation approach was used (Methods).

Firstly, we considered the genetic perturbations and assessed the capacity of TFs and K/Ps activities to predict the changes of a given metabolite across the panel of knockouts (Fig 3B, metabolites). This would be, for example, changes in concentration of glutamine across the knockout conditions. We evaluated the capacity of the models by correlating the independently predicted fold-changes to the observed ones. This procedure was performed for each metabolite and the results were summarized as correlation distributions. The metabolite variation across the different conditions were generally poorly predicted using either the TFs or K/P activities, displaying median correlations close to zero (Fig 3B). This did not change when we used the growth corrected data. We then measured how well the models predict the changes in all metabolites in a given condition (Fig 3B, conditions). This tests the capacity to, for example, predict the changes of all metabolite changes in HOG1 knockout. Overall, we obtained similar results as with the metabolites analysis, one difference is the improvement in predictive power considering K/Ps activities. However, this increase is mostly lost when we use the growth corrected data.

A similar analysis as with genetic perturbations was applied to the dynamic experiments to estimate the metabolic variation. The trained models displayed in general higher predictive power than the genetic perturbations (Fig 3C). Overall, in the dynamic nitrogen experiments, TFs displayed better agreement between measured and predicted metabolite fold-changes than K/Ps, across metabolites (Fig 3C, metabolites) and across conditions (Fig 3C, conditions). Also, models trained with growth normalised activities obtained similar results to non-normalised data-sets. The metabolic changes in the salt and pheromone experiment could be reasonably explained using the K/P activities across metabolites (Pearson’s r = 0.32) (Fig 3C, metabolites) and conditions (Pearson’s r = 0.41) (Fig 3C, metabolites). These were generally worse than the nitrogen experiment, and could be a consequence of the lower number of significantly changing metabolites when compared to the nitrogen experiments.

The predictive difference between growth normalised and non-normalised K/Ps activities in the genetic perturbations (Fig 3B, conditions) suggest that associations important to predict a new condition are generally dependent on global growth effects, and thereby likely to be indirect. Furthermore, the different predictive power between TFs and K/Ps on the dynamic nitrogen experiments suggest that changes in TF activities are more predictive of metabolic changes. Nevertheless, one needs to consider that very different technologies are used to measure the underlying data-sets, i.e. transcriptomics and phosphoproteomics and this may impact the predictive power of the data-sets.

### Inferring putative regulatory protein-metabolites interactions

Considering that the metabolic predictions based on time-resolved experiments partially circumvented indirect effects and displayed the best predictive power (Fig 3B and 3C) we decided to focus only on these data-sets, covering a total of 26 different conditions, for the inference of protein-metabolite regulatory interactions. Moreover, since growth has been shown to possibly act as a confounding effect (Fig 3B) we only used the data-set with growth normalised for the nitrogen metabolism experiments. We also considered TFs and K/Ps separately and searched for putative regulatory associations with the metabolite changes.

**Fig 4:**
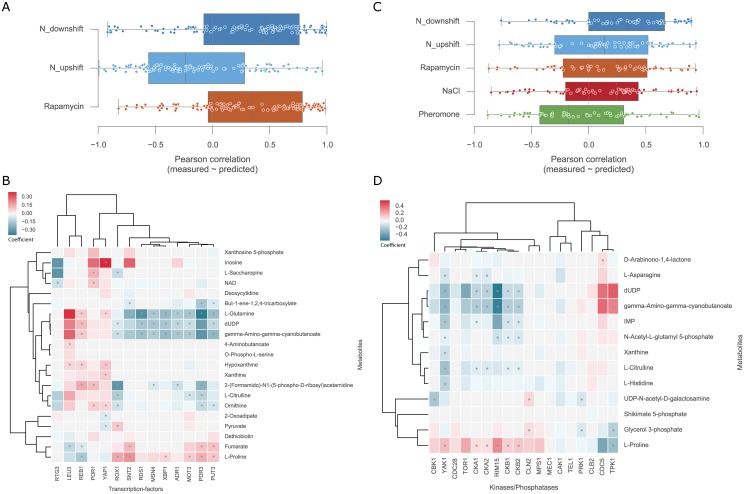
Overview of the putative protein-metabolite regulatory interactions. (A) Distribution of the metabolite predicted and measured correlations using 3-fold cross-validation leaving each condition out at a time using the TFs activities scores. (B) Heatmap of the TFs-metabolites associations where values represent the averaged coefficients. (C) Correlation distributions between predicted and measured using K/P activities with 5-fold cross-validation leaving each condition out at a time. (D) Heatmap of the K/Ps-metabolites associations where values represent the averaged coefficients. Coefficients distributions are calculated using a bootstrap cross-validation randomly leaving 20% of all the samples out. This procedure is performed twenty times and the coefficients are then averaged. Asterisks (*) identify significant, FDR < 5%, Pearson correlations between the activity profiles and the metabolite fold-change across all conditions.

We started by investigating the capacity of the TFs activities to estimate the metabolites fold-changes in each nitrogen related perturbation. To this end, we used a learning procedure, analogous to the one used before, but instead of LOO, a three-fold cross-validation was used to leave each of the environmental perturbations out at a time (see Methods). This was performed independently for each metabolite and the agreement between the measured and predicted values was calculated using Pearson correlation coefficients (Fig 4A). Consistently with the previous analysis, a large fraction of the metabolites were well predicted in downshift and rapamycin conditions. The best performances are obtained in the nitrogen downshift and the rapamycin experiments with similar median correlations. This could be expected since these are related conditions and the relationships learned from one may more readily apply to the other. Then, we considered only the best predicted metabolites (S3A Fig) and explored putative protein-metabolite associations using all the three nitrogen conditions together with bootstrapped linear regression models (see Methods). The associations were estimated 20 times with 80% of the samples randomly selected, therefore generating 20 coefficients for each TF-metabolite association. The average of the TF-metabolite coefficients represents a confidence score on the association (Fig 4B).

From the reported associations, LEU3 involved in the biosynthesis of leucine is positively associated with several metabolites involved in the biosynthesis of amino-acids, e.g. L-glutamine, L-citrulline, ornithine [43,44]. Recent work showed that gene deletion of LEU3 in yeast leads indeed to decreased L-glutamine abundance [45]. Also, the involvement of PUT3 in the proline utilisation pathways and its positive association with L-proline is captured by the linear model coefficients [46–48]. These results seem to confirm that the regulatory interactions found are biologically relevant, although they can be a result of direct or indirect associations. For example, a direct interaction can occur if a TF regulates the expression of metabolic enzymes and thereby controls directly metabolite concentration. The association can occur in the opposite direction where metabolites can directly regulate the activity of TFs. In contrast, indirect associations can be established, for instance, if metabolite changes are a consequence of downstream effects of TFs or if a cell state results in changes of both TF activity and metabolite concentration independently. In order to study this, we firstly identified the enzymes that use or produce each measured metabolite and considered a list of known TF-target proteins (from our assembled TF regulatory network), TF-gene genetic interactions (from BioGRID [49–51]) or TF-gene functional interactions (from STRING [52]) (see Methods). We then searched for enrichment of known TF-target, TF-gene genetic and functional associations among the top predicted TF-enzyme-metabolite interactions (S4A Fig). No significant association was found. We also note that the variation in TFs activities are almost fully explained by the first PC that captures 85.6% of the total variance in the data (S2 Fig). Furthermore, TFs activities showed similar profiles within TOR1 inhibition conditions, nitrogen downshift and rapamycin, and opposing profiles in TOR1 activation condition, nitrogen upshift. Hence, this shows lack of specificity in the gene expression response and can partially explain the limited capacity to identify direct associations with metabolites. However, regressing-out the first principal component from the TFs activity scores and from the metabolomics measurements did not improve the enrichment in direct TF-target associations. These findings support the idea that although the TFs activities are predictive of metabolic changes these relationships are likely to be indirectly due to changes in cellular states or via transcriptional regulation of genes that are not those immediately in the vicinity of the associated metabolites.

For the K/P-metabolite associations we used all five dynamic perturbations: nitrogen upshift, nitrogen downshift, rapamycin, NaCl and pheromone (Fig 4C). With the exception of the pheromone the other conditions showed similar median correlations between the measured and predicted metabolites fold-changes. However, the performance is overall lower than for the leave-one-out test (Fig 3C), as would be expected from a more stringent evaluation. The top predicted metabolites were selected (S3B Fig) and an analogous approach used for the TFs was used to identify K/P-metabolite associations (see Methods) (Fig 4D).

RIM15 and TPK1 displayed the strongest associations with the metabolites and these play a key role in the regulation of the cellular growth and their adaptation to nutrient availability [53–55]. TPK1 inhibits the activity of RIM15 to regulate cell cycle, thus this justifies that both display opposite associations (Pearson’s r -0.92, *p*-value < 8.6e-6). Furthermore, RIM15 is inhibited by TOR1 [55,56] and considering that L-proline is a poor nitrogen source leading to decreased TOR1 activity, this is consistent with the positive association between RIM15 and L-proline, and that TPK1 displays the inverse. Of note, TOR1 and RIM15 display similar metabolite relationships despite their inverse biological association, this happens because TOR1 activity is wrongly estimated due to lack of robustly measured targets. This is emphasised with the non-significant negative correlation between TOR1 and RIM15 activity scores (Pearson’s r -0.17, p-value < 3.98e-1). CKB1 and CKB2 positive associations with L-proline are confirmed by an independent study where gene deletions of these kinases are associated with depletion of L-proline [45]. Moreover, we could validate the negative association between YAK1 and L-asparagine, where knocking out YAK1 leads to an increase of L-asparagine [45]. The associations may be direct causal K/P-enzyme-metabolite relationships, but they could also be indirect or in the opposite direction where a metabolite change impacts kinase activity. We performed an enrichment analysis similar to the one described before, but now considering from BioGRID genetic and physical interactions. For each K/P-gene network we tested for enrichment of true interactions in the top-predicted K/P-enzyme-metabolite associations (S4B Fig). We observed a significant but weak enrichment for functional interactions (AROC = 0.63, S4B Fig) and direct K/P-target relationships (AROC = 0.61, S4B Fig). This significant enrichment in known K/P-target associations contrasts to the inferred TF-metabolites associations above. Specifically, from 16 functional interactions reported in STRING overlapping in our set of inferred associations half of those displayed a positive absolute coefficient. These results suggest that the retrieved associations contain some direct K/P-target relationships.

TFs-metabolites and K/Ps-metabolites associations can also be taken together to elucidate associations between K/Ps and TFs. For example, TPK1 kinase inhibits the activity of TF ADR1 [54] and these have inverse associations considering the five top predicted metabolites that they share (Pearson’s r -0.92, *p*-value < 2.6e-2). Also, YAK1 is required for full activation of MSN2/4 TFs [55] and this association is visible, although not significant, between MSN4 and YAK1 metabolites associations (Pearson’s r 0.8, *p*-value < 1.1e-1), for example, both have negative associations with dUDP and positive associations with L-Proline. However, this type of analysis is limited to the number of metabolites that can be well predicted with the TFs and K/Ps activities.

In summary, the estimated activity of TFs and K/Ps were capable of building predictive models to infer the metabolic adaptations of previously unseen conditions across different dynamic experiments. Specifically, TFs activities provided better predictive power than K/Ps, although K/Ps-metabolite associations are more likely to represent previously reported interactions.

## Discussion

Signal transduction is an important cellular mechanism that allows cells to sense and respond to environmental cues. These mediate intracellular adaptations by regulating a variety of biological processes, including metabolism and gene expression. Thereby interactions among different biological processes occur and are very important to coordinate the whole phenotype of the cell. Nevertheless, the systematic identification and functional annotation of these regulatory interactions is still a challenge. Experimental data-sets covering different omics in similar conditions are becoming more available and it is likely that analyses like the one we propose here will be useful to systematically explore these regulatory events.

The key novelty of the approach proposed here is that regulatory interactions are inferred from estimated activity of TFs and K/Ps, which are difficult to measure directly. This provides the possibility of considering the activity profile of regulatory proteins important for the experimental conditions at hand which has not been considered in previous studies of metabolism using phosphoproteomics and transcriptomics.

Our results show that it is possible to use K/Ps and TFs activities to predict changes of several metabolites in time-resolved experiments. However, the predictive power does not extend to all conditions. For example, the models trained with K/Ps activities showed limited capacity in the pheromone perturbation experiment. This can arise from the higher technical variability in the data obtained and also be due to lower number of regulated metabolites. Additionally, the regulatory interactions are often condition specific. As such, if the proteins that are important to regulate the nitrogen or osmotic related conditions are not used for the pheromone response, then the associations learned cannot be predictive of the pheromone induced metabolic changes. Interestingly, protein-metabolite interactions inferred from the genetic perturbations experiment displayed poor predictive power to estimate the metabolic changes of a new condition, in contrast to the dynamic experiments (Fig 3B and 3C). This suggests that time-resolved experiments provide a more efficient design to infer regulatory associations by circumventing general confounding effects that can be seen in the steady-state.

Nevertheless, while the protein-metabolite interactions that we infer provide reasonable power to predict metabolic changes (Fig 4A and 4C) of unseen conditions the predicted regulator-enzyme-metabolite interactions are not strongly enriched in previously regulatory interactions. Some features, particularly kinases or phosphatases activities, such as RIM15 and TPK1, were important features to estimate metabolite fold-changes. This reassuringly assesses that the estimated protein activity profiles are biologically relevant and useful for inferring metabolic adaptation in novel conditions. The time-resolved metabolomics experiment under salt and pheromone resulted in only moderate metabolic changes, when compared to the nitrogen conditions. This smaller variation may explain the lower power in identifying regulator-metabolite associations in these conditions. We believe that this emphasises the importance of designing experiments that adequately perturb both signalling and metabolism, without possible confounding effects, such as CDC28 inhibition. Another possible limitation of this approach is that, while we used comprehensive resources, we only considered prior knowledge of reported K/Ps-substrate and TFs-gene regulatory interactions. Furthermore, the lack of missing values in transcriptomics data-sets provides increased robustness to TFs activities when compared to K/Ps activities. Nevertheless, both protein activity profiles showed comparable predictive power, thus partially guaranteeing that the existing bias does not penalise greatly the K/Ps.

The increasing number of recorded interactions for regulators will provide important information to expand the coverage of TFs and K/Ps for which it is possible to estimate activities and increase the robustness of the estimated activity. The generic characteristic of the approach used to infer regulatory interactions allows it to be easily expanded to integrate other types of information and thereby augment its predictive power to infer causal and direct protein-metabolite interactions. For example, to account for other confounding effects, such as cell cycle as a covariate and thereby remove it as a possible source of interactions. Furthermore, other types of biological measurements can also be integrated, for example, protein abundance. This can provide information into other regulatory mechanisms but also provide information to possible associations between the different regulatory processes, for instance, phosphoproteomics measurements are intrinsically dependent on protein abundance.

In this study we demonstrated the utility of phosphoproteomics and transcriptomics data-sets to estimate the enzymatic activity of K/Ps and TFs, respectively. The estimated activities recapitulated several previously expected regulatory events, such as HOG1 and PBS2 responses to osmotic stress, and RIM15 and MSN2/4 activation under TOR1 inhibition. This results emphasise the usefulness of this approach to explore functional implications in regulatory proteins. Our results also showed that activity profiles are informative features to estimate metabolite changes in dynamic experiments. Interestingly, the same was not visible across a large panel of K/Ps knockouts, supporting the idea that time-resolved experiments are a better experimental design for the identification of causal regulatory interactions. We expanded on previous work by developing a novel and rigorous framework to identify regulatory associations between the estimated activities and metabolite changes. Rigorous analysis of the putative TFs-metabolite and K/Ps-metabolite associations occurring in the dynamic experiments revealed that despite their regulatory implication these are most likely indirect. Further confirmation of these results with the integration of other experimental data-sets will provide deeper insights into the regulatory events mediating the metabolic phenotype.

## Materials and Methods

### Strain, growth and sample preparation

The *Saccharomyces cerevisiae* strain used for the salt and pheromone dynamic experiments was BY4741 as in Vaga et al. [22,23], this strain is provided with a CDC28-as allele that can be directly inhibited by means of 1-NA-PP1, the ATP analog “PP1 analog 8”. Cells were grown in 500-ml shake flasks at 30°C in 50 ml SD medium to an OD600 of 0.6. The ATP analog was added to a final concentration of 10μM. One hour after CDC28 inhibition cells were perturbed with NaCl to a final concentration of 0.4M or pheromone to a final concentration of 1 μM. Cells were extracted by vacuum-filtering culture aliquots on a 0.45 μm pore size nitrocellulose filter (Millipore). The filter was immediately transferred to 3 ml 2:2:1 MeOH/AcN/ddH2O precooled at -30°C. Samples for LC-MS/MS were supplemented with 200 μl uniformly-labeled 13C *E*. *coli* extract as internal standard and dried completely in a vacuum centrifuge (Christ-RVC 2–33 CD plus, Kuehner AG, Birsfelden, Switzerland). The dried extracts were resuspended in 100 μl MilliQ water before analysis.

### Acquisition of intracellular metabolite levels

Targeted metabolomics was performed by LC-MS/MS as described before [24]. The mass-spectrometer was operated in negative mode. Data acquisition and peak integration were performed with the Xcalibur software version 2.07 SP1 (Thermo Fisher Scientific) and in-house integration software. Metabolite peak areas were normalized to uniformly-labeled 13C internal standards. Samples were supplemented with 54 carbon labeled extracts for which both the labeled and unlabeled spectral mass peaks were manually identified and curated. The manual quality assessment of the peaks lead us to retain 26 metabolites which could be reliably identified from the sample spectra.

Untargeted metabolomics was performed by direct flow double injection of extracts on an Agilent 6550 series quadrupole TOF MS operated in negative mode. In total 11,190 ions were detected across all samples. The detected ions were then mapped against an existing ion mass library of metabolites generated from the yeast genome-scale model iMM904 [26]. 452 out of 647 ion entries in the library were identified in our data-set and these were kept for the downstream analysis. Considering that natural modifications can occur and change metabolite mass we used a rigorous annotation of the detected ions to only consider deprotonated metabolites, therefore reducing the number of detected and annotated ions to 196. These filtering steps allowed us to consider only highly confident annotated ions, which mapped to 270 yeast metabolites (S1 Table). The overall performance of the untargeted metabolomics was compared to the targeted metabolomics (Fig 1D). High concentrations of salt in the NaCl perturbation experiment resulted in a strong effect on the ion matrix in the QTOF-MS measurements. To prevent this matrix effect from affecting data analysis we normalised the data to the second time-point (25 seconds) instead of the 0 seconds timepoint.

Statistical significance of the ion fold-changes for the QTOF-MS measurements was estimated with a two-sided t-test followed by multiple hypothesis correction with false-discovery rate. The list was then filtered and only ions with and an absolute fold-change higher than 1 were considered, resulting in 33 ions.

### Compendium of yeast data-sets

For K/Ps knockouts in yeast, a total of 3,011 transcripts were measured across 1,484 deletion mutants, comprising approximately 26.4% of all protein-coding genes in yeast [57]. Phosphoproteomics profiles of 125 K/P knockouts, as compared to a wild-type strain, were acquired using label free mass-spectrometry (LC-MS/MS) measuring 4,263 unique single phosphorylated phosphosites in at least one condition. Intracellular measurements of metabolites were obtained during the exponential growth phase and analyzed using non-targeted direct injection and time-of-flight mass-spectrometry (QTOF-MS). In total, 1,698 unique ions were detected across 118 kinases/phosphatases knockouts. Metabolomics and phosphoproteomics data-sets overlap in 115 knock-out conditions, and metabolomics intersects the transcriptomics in 45 knock-out conditions.

Dynamic perturbations to nitrogen metabolism and TOR signalling were captured in a time frame from 0 to 79 minutes, where 0 minutes represents the unperturbed state. Transcriptomics measurements covered 5,620 transcripts across all the time-points of the conditions. Phosphoproteomics captured the profile of 1,660 single phosphorylated phosphosites (84.8% serines, 14.2% threonines and 1.0% tyrosines) over the same time-points. Given the lack of complete coverage, only 50.8% of the whole matrix is measured. Intracellular metabolomics were acquired with QTOF-MS and quantified a total of 146 ions, after quality filtering, across all conditions and time-points.

### Activity inference method

Kinases/phosphatases and transcription factor activities were estimated using GSEA approach [36] and statistical significance was calculated against a null hypothesis generated by randomising 1000 times the regulator reported targets [36]. Activity scores were calculated using the log10 of the empirical *p*-value and signed according to the direction of the enrichment, for example, regulators enriched towards negative fold-changes have a negative score. K/Ps target phosphosites were extracted from PhosphoGrid [34]. Specificities for a total of 177 transcription factors were collected in form of a position weight matrices (PWMs) from JASPAR [58]. Weight matrices were trimmed to remove consecutive stretches of low information content (<0.2) on either end. The log-scoring scheme defined in [59], was used to score potential target sequences against weight matrices. The log score is normalised to the best and worst matching sequence to the weight matrix, resulting in a value that lies between 0 and 1, where 1 denotes strong binding to the matrix and 0 denotes no binding. Genome wide gene expression profiles for 837 gene-knockout strains were collected from three studies [5,60,61], of which 148/837 were a known transcription factor with a defined specificity weight matrix. Studies provided either a Z-score or *p*-value for each gene as a measure of over or under-expression, relative to the distribution of values for all genes. Two-tailed p-values were computed from Z-scores when a p-value was not provided [61]. In cases where TF knockout was repeated between studies, the lowest p-value for each gene was used. ChIP-ChIP tracks for 355 proteins were collected from four studies [62–65], via the Saccharomyces genome database [66]. 144/355 of proteins were transcription factors with a defined specificity weight matrix. The TF-gene network was then defined as all TF-gene pairs with a p-value below 0.01 and contained a ChIP-ChIP region upstream of the regulated gene, which scored highly against the weight matrix of the TF (normalised logscore>0.9).

### Linear regression methods for estimating metabolic changes

Python module Sklearn [67] version 0.16.1 was used to perform linear regression analysis and default parameters were used unless stated otherwise. Linear models with combined L1 and L2 regularization, Elastic Net, was used with the l1_ratio of 0.5. Elastic net regularization simplifies the complexity of the model by removing the least important features, similar to Lasso regularisation, but also considering a L2 regularization, similar to Ridge, to avoid random feature elimination when collinearity exists among the features.

To infer the predictive power within each data-set across all the measured ions (Fig 3B), for each metabolomics data-set, ions displaying low variation across the samples were discarded by considering only those that showed a standard deviation higher than 0.4. KPs activities were filtered to only consider kinases or phosphatases with an activity score estimated in at least 75% of the samples of each data-set, the remaining missing values were replaced with zeros for the machine learning approaches. For the Elastic net regressions different alphas were tested and an alpha of 0.01 obtained the best overall performance, therefore this was used in all the models. For each metabolite a leave-one-out cross-validation was used, thus all but one sample were used to train the linear regression model and then the test sample was used to estimate the metabolite fold-change. Performing this systematically across all metabolites and conditions generated a predicted matrix for which each value is estimated independently. The agreement between the measured and predicted ions fold-changes was calculated with Pearson correlation coefficients across rows (ions) and columns (conditions).

### Linear regression models to predict K/Ps-metabolites and TFs-metabolites associations

Protein-metabolite associations were inferred only using the time-resolved metabolomics data-sets. For the TFs activities only the nitrogen metabolism perturbations were used considering that no transcriptomics data was available for the NaCl/Pheromone perturbations. For this analysis no filtering was applied in the metabolomics data-sets and K/Ps activities were filtered as before to consider only those consistently measured and estimated across 75% of the conditions. The capacity of predicting each ion fold-change in each condition was tested using k-fold cross-validation, where each fold corresponds to the time-points measured in each condition, thus 3 and 5 folds were used for the TFs and KPs activities, respectively. Elastic net models were used and the alpha was estimated using a bootstrap approach of ten iterations leaving out 20% of the samples. A range of 100 alphas was considered as default by the Sklearn python module [67]. Train and test features, TFs and KPs activities, were standardised, and the observed variables, metabolomics, were centered before training the linear model. The predictive power of each ion in each condition was estimated by using the k-fold models, inferring the agreement between predicted and measured in the left-out condition using Pearson coefficient and coefficient of determination metrics. The top predicted ions were those that displayed a Pearson correlation *p*-value lower than 0.05 and an coefficient of determination higher than zero.

For the top predicted ions the feature importance was estimated using all the conditions together with two bootstraps. The first bootstrap was 20 iterations and leaves out 20% of the samples out, for each iteration an inner bootstrap with 10 iterations leaving out another 20% of the data is performed to estimate the alpha of the Elastic net. This estimates 20 coefficients for each feature-metabolite association. As before, train features and observations are standardised and centered. The most important features per ion are estimated by taking the median of the coefficients and the Pearson correlation between the protein activity and the ion fold-change.

### Data and analysis code availability

All data analysis was performed in Python (v 2.7.10) and all the code, preprocessed data-sets and generated plots are openly available in github under the GNU general public license version 3 in the following URL https://github.com/saezlab/yeast_phospho. All plotting was performed using Python modules Matplotlib version 1.4.3 [68] and Seaborn version 0.7.0 [69].

## Supporting Information

S1 Fig Overlap of the features measured across the three different data-sets, i.e. genetic perturbations, nitrogen metabolism and NaCl + pheromone. a) identifies the overlap between the TF activities, b) between the K/P activities and c) between the metabolite ions measured. For the overlap it was considered only TFs and K/Ps for which it was possible to estimate their activity in at least 75% of the samples in each data-set.
(TIF)

S2 Fig Principal component analysis of data-sets and correlation with relative growth rate. The principal component with higher absolute correlation coefficient was picked and plotted.
(TIF)

S3 Fig List of top predicted metabolites using a) TFs activities and b) K/Ps activities. List of metabolites that displayed a positive coefficient of determination and significant Pearson correlation between the measured and predicted fold-changes across the different conditions.
(TIF)

S4 Fig ROC-curve analysis of the average feature coefficients. True-positive tables were built considering the specified resources.
(TIF)

S1 Table Salt and pheromone metabolomics experiments in yeast and metabolites annotation. (XLSX)

S2 Table Kinases/phosphatases and transcription factor activity scores. (XLSX)

S3 Table Protein-metabolites interactions betas. (XLSX)

## References

[1] Chubukov V, Gerosa L, Kochanowski K, Sauer U. Coordination of microbial metabolism. Nat Rev Microbiol. 2014;12: 327–340. 10.1038/nrmicro3238 24658329

[2] Herrgård MJ, Swainston N, Dobson P, Dunn WB, Arga KY, Arvas M, et al A consensus yeast metabolic network reconstruction obtained from a community approach to systems biology. Nat Biotechnol. 2008;26: 1155–1160. 10.1038/nbt1492 18846089 PMC4018421

[3] Daran-Lapujade P, Rossell S, van Gulik WM, Luttik MAH, de Groot MJL, Slijper M, et al The fluxes through glycolytic enzymes in Saccharomyces cerevisiae are predominantly regulated at posttranscriptional levels. Proc Natl Acad Sci U S A. 2007;104: 15753–15758. 10.1073/pnas.0707476104 17898166 PMC2000426

[4] Bodenmiller B, Wanka S, Kraft C, Urban J, Campbell D, Pedrioli PG, et al Phosphoproteomic Analysis Reveals Interconnected System-Wide Responses to Perturbations of Kinases and Phosphatases in Yeast. Sci Signal. AAAS; 2010;3: rs4–rs4. 10.1126/scisignal.2001182 PMC3072779 21177495

[5] Kemmeren P, Sameith K, van de Pasch LAL, Benschop JJ, Lenstra TL, Margaritis T, et al Large-scale genetic perturbations reveal regulatory networks and an abundance of gene-specific repressors. Cell. Elsevier Inc.; 2014;157: 740–752. 10.1016/j.cell.2014.02.054 24766815

[6] Schulz JC, Zampieri M, Wanka S, von Mering C, Sauer U. Large-scale functional analysis of the roles of phosphorylation in yeast metabolic pathways. Sci Signal. AAAS; 2014;7: rs6. 10.1126/scisignal.2005602 25429078

[7] Oliveira AP, Ludwig C, Picotti P, Kogadeeva M, Aebersold R, Sauer U. Regulation of yeast central metabolism by enzyme phosphorylation. Mol Syst Biol. Nature Publishing Group; 2012;8. 10.1038/msb.2012.55 PMC3531909 23149688

[8] Zelezniak A, Sheridan S, Patil KR. Contribution of network connectivity in determining the relationship between gene expression and metabolite concentration changes. PLoS Comput Biol. 2014;10: e1003572 10.1371/journal.pcbi.1003572 24762675 PMC3998873

[9] Patil KR, Nielsen J. Uncovering transcriptional regulation of metabolism by using metabolic network topology. Proc Natl Acad Sci U S A. 2005;102: 2685–2689. 10.1073/pnas.0406811102 15710883 PMC549453

[10] Gerosa L, Haverkorn van Rijsewijk BRB, Christodoulou D, Kochanowski K, Schmidt TSB, Noor E, et al Pseudo-transition Analysis Identifies the Key Regulators of Dynamic Metabolic Adaptations from Steady-State Data. Cell Systems. Elsevier; 2015;1: 270–282. 10.1016/j.cels.2015.09.008 27136056

[11] Oliveira AP, Dimopoulos S, Busetto AG, Christen S, Dechant R, Falter L, et al Inferring causal metabolic signals that regulate the dynamic TORC1-dependent transcriptome. Mol Syst Biol. EMBO Press; 2015;11: 802. 10.15252/msb.20145475 PMC4422559 25888284

[12] Oliveira AP, Ludwig C, Zampieri M, Weisser H, Aebersold R, Sauer U. Dynamic phosphoproteomics reveals TORC1-dependent regulation of yeast nucleotide and amino acid biosynthesis. Sci Signal. AAAS; 2015;8: rs4. 10.1126/scisignal.2005768 25921291

[13] Daran-Lapujade P, Jansen MLA, Daran J-M, van Gulik W, de Winde JH, Pronk JT. Role of transcriptional regulation in controlling fluxes in central carbon metabolism of Saccharomyces cerevisiae. A chemostat culture study. J Biol Chem. 2004;279: 9125–9138. 10.1074/jbc.M309578200 14630934

[14] Machado D, Herrgård M. Systematic evaluation of methods for integration of transcriptomic data into constraint-based models of metabolism. PLoS Comput Biol. Public Library of Science; 2014;10: e1003580. 10.1371/journal.pcbi.1003580 PMC3998872 24762745

[15] Yugi K, Kubota H, Toyoshima Y, Noguchi R, Kawata K, Komori Y, et al Reconstruction of insulin signal flow from phosphoproteome and metabolome data. Cell Rep. 2014;8: 1171–1183. 10.1016/j.celrep.2014.07.021 25131207

[16] Cheng C, Alexander R, Min R, Leng J, Yip KY, Rozowsky J, et al Understanding transcriptional regulation by integrative analysis of transcription factor binding data. Genome Res. 2012;22: 1658–1667. 10.1101/gr.136838.111 22955978 PMC3431483

[17] Schacht T, Oswald M, Eils R, Eichmüller SB, König R. Estimating the activity of transcription factors by the effect on their target genes. Bioinformatics. 2014;30: i401–7. 10.1093/bioinformatics/btu446 25161226 PMC4147899

[18] Casado P, Rodriguez-Prados J-C, Cosulich SC, Guichard S, Vanhaesebroeck B, Joel S, et al Kinase-substrate enrichment analysis provides insights into the heterogeneity of signaling pathway activation in leukemia cells. Sci Signal. 2013;6: rs6 10.1126/scisignal.2003573 23532336

[19] Mischnik M, Sacco F, Cox J, Schneider H-C, Schäfer M, Hendlich M, et al IKAP: A heuristic framework for inference of kinase activities from Phosphoproteomics data. Bioinformatics. 2016;32: 424–431. 10.1093/bioinformatics/btv699 26628587

[20] Brückner S, Kern S, Birke R, Saugar I, Ulrich HD, Mösch H-U. The TEA transcription factor Tec1 links TOR and MAPK pathways to coordinate yeast development. Genetics. 2011;189: 479–494. 10.1534/genetics.111.133629 21840851 PMC3189810

[21] Saito H, Posas F. Response to hyperosmotic stress. Genetics. 2012;192: 289–318. 10.1534/genetics.112.140863 23028184 PMC3454867

[22] Vaga S, Bernardo-Faura M, Cokelaer T, Maiolica A, Barnes CA, Gillet LC, et al Phosphoproteomic analyses reveal novel cross-modulation mechanisms between two signaling pathways in yeast. Mol Syst Biol. 2014;10: 767 10.15252/msb.20145112 25492886 PMC4300490

[23] D’Aquino KE, Monje-Casas F, Paulson J, Reiser V, Charles GM, Lai L, et al The Protein Kinase Kin4 Inhibits Exit from Mitosis in Response to Spindle Position Defects. Mol Cell. 2005;19: 223–234. 10.1016/j.molcel.2005.06.005 16039591

[24] Buescher JM, Moco S, Sauer U, Zamboni N. Ultrahigh performance liquid chromatography-tandem mass spectrometry method for fast and robust quantification of anionic and aromatic metabolites. Anal Chem. American Chemical Society; 2010;82: 4403–4412. 10.1021/ac100101d 20433152

[25] Fuhrer T, Heer D, Begemann B, Zamboni N. High-Throughput, Accurate Mass Metabolome Profiling of Cellular Extracts by Flow Injection–Time-of-Flight Mass Spectrometry. Anal Chem. 2011;83: 7074–7080. 10.1021/ac201267k 21830798

[26] Mo ML, Palsson BO, Herrgård MJ. Connecting extracellular metabolomic measurements to intracellular flux states in yeast. BMC Syst Biol. BioMed Central Ltd; 2009;3: 37. 10.1186/1752-0509-3-37 PMC2679711 19321003

[27] Park JO, Rubin SA, Xu Y-F, Amador-Noguez D, Fan J, Shlomi T, et al Metabolite concentrations, fluxes and free energies imply efficient enzyme usage. Nat Chem Biol. 2016; 10.1038/nchembio.2077 PMC4912430 27159581

[28] Kanshin E, Bergeron-Sandoval L-P, Isik SS, Thibault P, Michnick SW. A cell-signaling network temporally resolves specific versus promiscuous phosphorylation. Cell Rep. 2015;10: 1202–1214. 10.1016/j.celrep.2015.01.052 25704821

[29] Mitchell A, Wei P, Lim WA. Oscillatory stress stimulation uncovers an Achilles’ heel of the yeast MAPK signaling network. Science. 2015; 10.1126/science.aab0892 PMC4721531 26586187

[30] Hohmann S. Osmotic stress signaling and osmoadaptation in yeasts. Microbiol Mol Biol Rev. Am Soc Microbiol; 2002;66: 300–372. 10.1128/MMBR.66.2.300-372.2002 PMC120784 12040128

[31] Merlini L, Dudin O, Martin SG. Mate and fuse: how yeast cells do it. Open Biol. 2013;3: 130008 10.1098/rsob.130008 23466674 PMC3718343

[32] Bardwell L. A walk-through of the yeast mating pheromone response pathway. Peptides. 2005;26: 339–350. 15690603 10.1016/j.peptides.2004.10.002 PMC3017506

[33] Beltrao P, Albanèse V, Kenner LR, Swaney DL, Burlingame A, Villén J, et al Systematic functional prioritization of protein posttranslational modifications. Cell. 2012;150: 413–425. 10.1016/j.cell.2012.05.036 22817900 PMC3404735

[34] Sadowski I, Breitkreutz B-J, Stark C, Su T-C, Dahabieh M, Raithatha S, et al The PhosphoGRID Saccharomyces cerevisiae protein phosphorylation site database: version 2.0 update. Database. 2013;2013: bat026 10.1093/database/bat026 23674503 PMC3653121

[35] Ochoa D, Jonikas M, Lawrence RT, El Debs B, Selkrig J, Typas A, et al An atlas of human kinase regulation. Mol Syst Biol. 2016;12: 888 10.15252/msb.20167295 27909043 PMC5199121

[36] Subramanian A, Tamayo P, Mootha VK, Mukherjee S, Ebert BL, Gillette MA, et al Gene set enrichment analysis: a knowledge-based approach for interpreting genome-wide expression profiles. Proc Natl Acad Sci U S A. 2005;102: 15545–15550. 10.1073/pnas.0506580102 16199517 PMC1239896

[37] Beck T, Hall MN. The TOR signalling pathway controls nuclear localization of nutrient-regulated transcription factors. Nature. 1999;402: 689–692. 10.1038/45287 10604478

[38] Schmidt A, Beck T, Koller A, Kunz J, Hall MN. The TOR nutrient signalling pathway phosphorylates NPR1 and inhibits turnover of the tryptophan permease. EMBO J. 1998;17: 6924–6931. 10.1093/emboj/17.23.6924 9843498 PMC1171040

[39] Pedruzzi I, Dubouloz F, Cameroni E, Wanke V, Roosen J, Winderickx J, et al TOR and PKA signaling pathways converge on the protein kinase Rim15 to control entry into G0. Mol Cell. 2003;12: 1607–1613. 14690612 10.1016/s1097-2765(03)00485-4

[40] Martin DE, Soulard A, Hall MN. TOR regulates ribosomal protein gene expression via PKA and the Forkhead transcription factor FHL1. Cell. 2004;119: 969–979. 10.1016/j.cell.2004.11.047 15620355

[41] O’Duibhir E, Lijnzaad P, Benschop JJ, Lenstra TL, van Leenen D, Groot Koerkamp MJA, et al Cell cycle population effects in perturbation studies. Mol Syst Biol. 2014;10: 732 10.15252/msb.20145172 24952590 PMC4265054

[42] Brauer MJ, Huttenhower C, Airoldi EM, Rosenstein R, Matese JC, Gresham D, et al Coordination of growth rate, cell cycle, stress response, and metabolic activity in yeast. Mol Biol Cell. 2008;19: 352–367. 10.1091/mbc.E07-08-0779 17959824 PMC2174172

[43] Friden P, Schimmel P. LEU3 of Saccharomyces cerevisiae activates multiple genes for branched-chain amino acid biosynthesis by binding to a common decanucleotide core sequence. Mol Cell Biol. 1988;8: 2690–2697. 3043190 10.1128/mcb.8.7.2690-2697.1988 PMC363479

[44] Nielsen PS, van den Hazel B, Didion T, de Boer M, Jørgensen M, Planta RJ, et al Transcriptional regulation of the Saccharomyces cerevisiae amino acid permease gene BAP2. Mol Gen Genet. 2001;264: 613–622. 11212916 10.1007/s004380000347

[45] Mülleder M, Calvani E, Alam MT, Wang RK, Eckerstorfer F, Zelezniak A, et al Functional Metabolomics Describes the Yeast Biosynthetic Regulome. Cell. 2016;167: 553–565.e12. 10.1016/j.cell.2016.09.007 27693354 PMC5055083

[46] Huang HL, Brandriss MC. The regulator of the yeast proline utilization pathway is differentially phosphorylated in response to the quality of the nitrogen source. Mol Cell Biol. 2000;20: 892–899. 10629046 10.1128/mcb.20.3.892-899.2000 PMC85206

[47] Axelrod JD, Majors J, Brandriss MC. Proline-independent binding of PUT3 transcriptional activator protein detected by footprinting in vivo. Mol Cell Biol. 1991;11: 564–567. 1986247 10.1128/mcb.11.1.564-567.1991 PMC359669

[48] Siddiqui AH, Brandriss MC. The Saccharomyces cerevisiae PUT3 activator protein associates with proline-specific upstream activation sequences. Mol Cell Biol. 1989;9: 4706–4712. 2689862 10.1128/mcb.9.11.4706-4712.1989 PMC363617

[49] Chatr-Aryamontri A, Breitkreutz B-J, Oughtred R, Boucher L, Heinicke S, Chen D, et al The BioGRID interaction database: 2015 update. Nucleic Acids Res. 2015;43: D470–8. 10.1093/nar/gku1204 25428363 PMC4383984

[50] Oughtred R, Chatr-aryamontri A, Breitkreutz B-J, Chang CS, Rust JM, Theesfeld CL, et al Use of the BioGRID Database for Analysis of Yeast Protein and Genetic Interactions. Cold Spring Harb Protoc. 2016;2016: db.prot088880. 10.1101/pdb.prot088880 PMC5975959 26729909

[51] Oughtred R, Chatr-Aryamontri A, Breitkreutz B-J, Chang CS, Rust JM, Theesfeld CL, et al BioGRID: A Resource for Studying Biological Interactions in Yeast. Cold Spring Harb Protoc. 2016;2016: db.top080754. 10.1101/pdb.top080754 PMC5975956 26729913

[52] Jensen LJ, Kuhn M, Stark M, Chaffron S, Creevey C, Muller J, et al STRING 8—a global view on proteins and their functional interactions in 630 organisms. Nucleic Acids Res. 2009;37: D412–6. 10.1093/nar/gkn760 18940858 PMC2686466

[53] Chavel CA, Caccamise LM, Li B, Cullen PJ. Global regulation of a differentiation MAPK pathway in yeast. Genetics. 2014;198: 1309–1328. 10.1534/genetics.114.168252 25189875 PMC4224168

[54] Conrad M, Schothorst J, Kankipati HN, Van Zeebroeck G, Rubio-Texeira M, Thevelein JM. Nutrient sensing and signaling in the yeast Saccharomyces cerevisiae. FEMS Microbiol Rev. 2014;38: 254–299. 10.1111/1574-6976.12065 24483210 PMC4238866

[55] Broach JR. Nutritional control of growth and development in yeast. Genetics. 2012;192: 73–105. 10.1534/genetics.111.135731 22964838 PMC3430547

[56] Swinnen E, Wanke V, Roosen J, Smets B, Dubouloz F, Pedruzzi I, et al Rim15 and the crossroads of nutrient signalling pathways in Saccharomyces cerevisiae. Cell Div. 2006;1: 3 10.1186/1747-1028-1-3 16759348 PMC1479807

[57] Byrne KP, Wolfe KH. The Yeast Gene Order Browser: combining curated homology and syntenic context reveals gene fate in polyploid species. Genome Res. 2005;15: 1456–1461. 10.1101/gr.3672305 16169922 PMC1240090

[58] Mathelier A, Zhao X, Zhang AW, Parcy F, Worsley-Hunt R, Arenillas DJ, et al JASPAR 2014: an extensively expanded and updated open-access database of transcription factor binding profiles. Nucleic Acids Res. 2014;42: D142–7. 10.1093/nar/gkt997 24194598 PMC3965086

[59] Wasserman WW, Sandelin A. Applied bioinformatics for the identification of regulatory elements. Nat Rev Genet. 2004;5: 276–287. 10.1038/nrg1315 15131651

[60] Hu Z, Killion PJ, Iyer VR. Genetic reconstruction of a functional transcriptional regulatory network. Nat Genet. 2007;39: 683–687. 10.1038/ng2012 17417638

[61] Chua G, Morris QD, Sopko R, Robinson MD, Ryan O, Chan ET, et al Identifying transcription factor functions and targets by phenotypic activation. Proc Natl Acad Sci U S A. 2006;103: 12045–12050. 10.1073/pnas.0605140103 16880382 PMC1567694

[62] Harbison CT, Gordon DB, Lee TI, Rinaldi NJ, Macisaac KD, Danford TW, et al Transcriptional regulatory code of a eukaryotic genome. Nature. 2004;431: 99–104. 10.1038/nature02800 15343339 PMC3006441

[63] Rhee HS, Pugh BF. Comprehensive genome-wide protein-DNA interactions detected at single-nucleotide resolution. Cell. 2011;147: 1408–1419. 10.1016/j.cell.2011.11.013 22153082 PMC3243364

[64] Tachibana C, Yoo JY, Tagne J-B, Kacherovsky N, Lee TI, Young ET. Combined global localization analysis and transcriptome data identify genes that are directly coregulated by Adr1 and Cat8. Mol Cell Biol. 2005;25: 2138–2146. 10.1128/MCB.25.6.2138-2146.2005 15743812 PMC1061606

[65] Venters BJ, Wachi S, Mavrich TN, Andersen BE, Jena P, Sinnamon AJ, et al A comprehensive genomic binding map of gene and chromatin regulatory proteins in Saccharomyces. Mol Cell. 2011;41: 480–492. 10.1016/j.molcel.2011.01.015 21329885 PMC3057419

[66] Christie KR, Weng S, Balakrishnan R, Costanzo MC, Dolinski K, Dwight SS, et al Saccharomyces Genome Database (SGD) provides tools to identify and analyze sequences from Saccharomyces cerevisiae and related sequences from other organisms. Nucleic Acids Res. 2004;32: D311–4. 10.1093/nar/gkh033 14681421 PMC308767

[67] Pedregosa F, Varoquaux G, Gramfort A, Michel V, Thirion B, Grisel O, et al Scikit-learn: Machine Learning in Python. J Mach Learn Res. JMLR.org; 2011;12: 2825–2830.

[68] Hunter JD. Matplotlib: A 2D Graphics Environment. Computing in Science Engineering. AIP Publishing; 2007;9: 90–95.

[69] Waskom M, Botvinnik O, Hobson P, Cole JB, Halchenko Y, Hoyer S, et al. seaborn: v0.5.0 (November 2014) [Internet]. ZENODO; 2014.

