# Supplementary figures and images for: Systematic Analysis of Transcriptional and Post-transcriptional Regulation of Metabolism in Yeast
Source: PLoS Comput Biol. 2017 Jan 10;13(1):e1005297. doi: 10.1371/journal.pcbi.1005297 (PMC5224888; doi:10.1371/journal.pcbi.1005297)

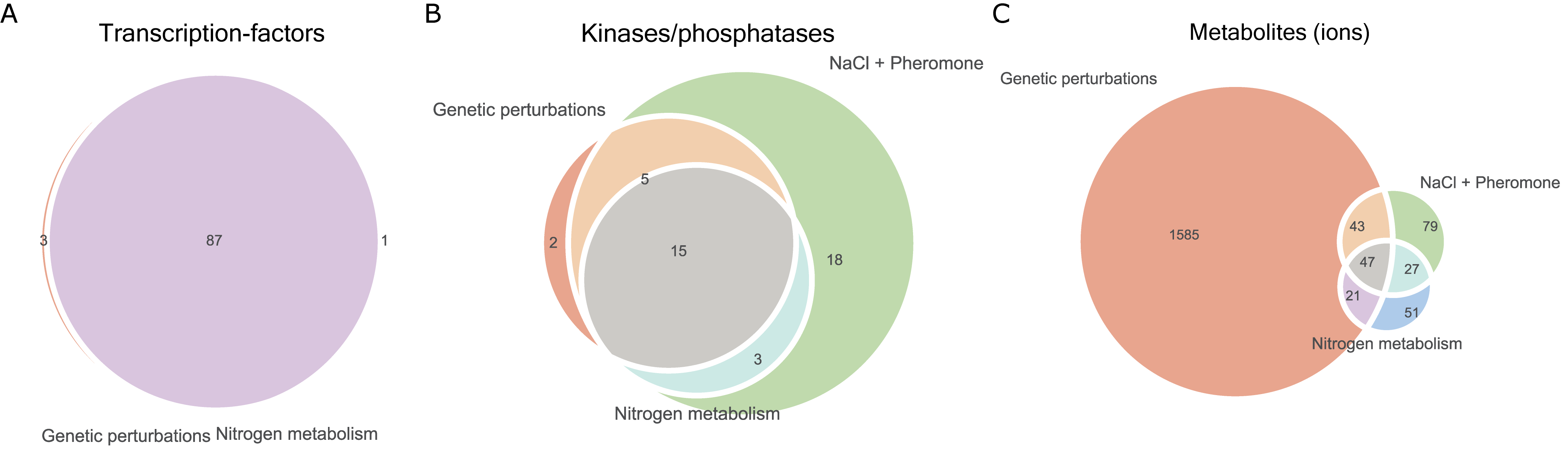

Supplement: S1 Fig — a) identifies the overlap between the TF activities, b) between the K/P activities and c) between the metabolite ions measured. For the overlap it was considered only TFs and K/Ps for which it was possible to estimate their activity in at least 75% of the samples in each data-set. (TIF) [file pcbi.1005297.s001.tif]

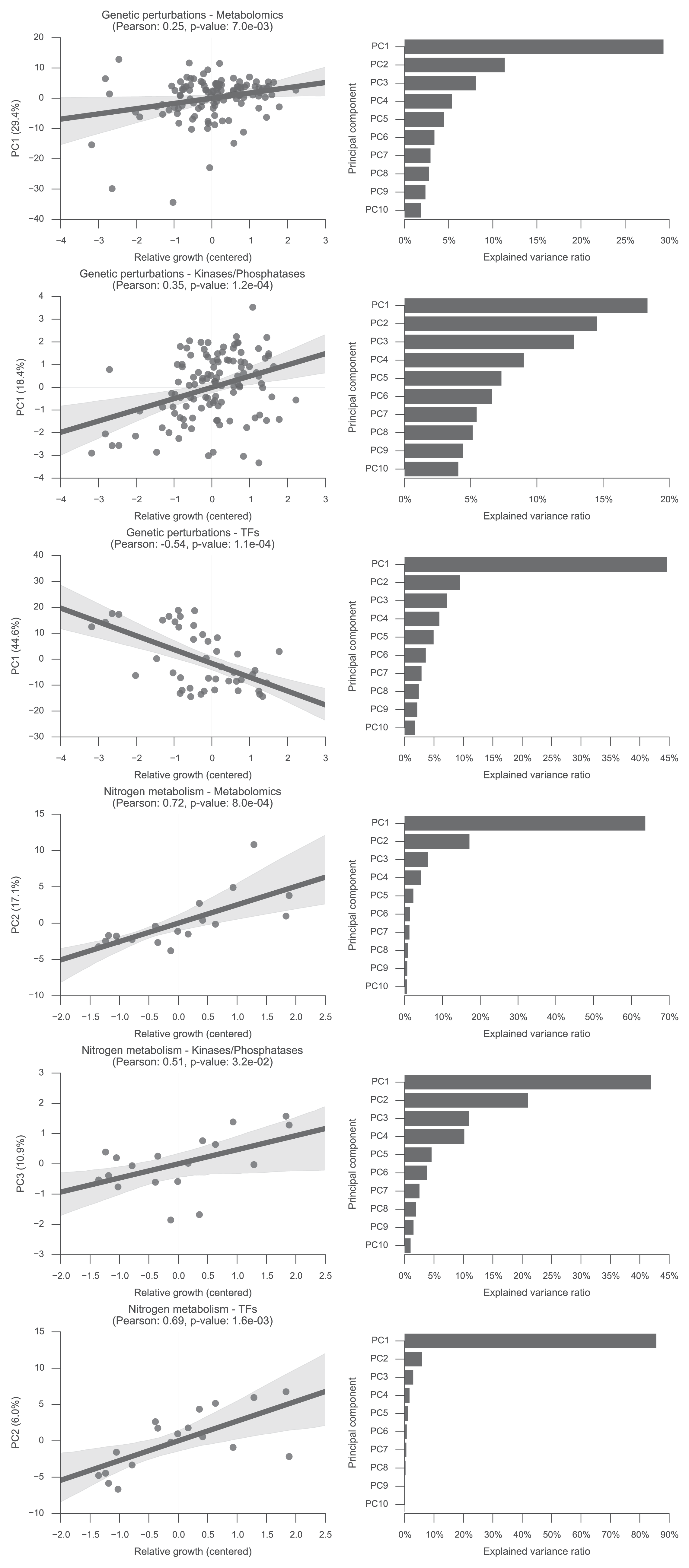

Supplement: S2 Fig — The principal component with higher absolute correlation coefficient was picked and plotted. (TIF) [file pcbi.1005297.s002.tif]

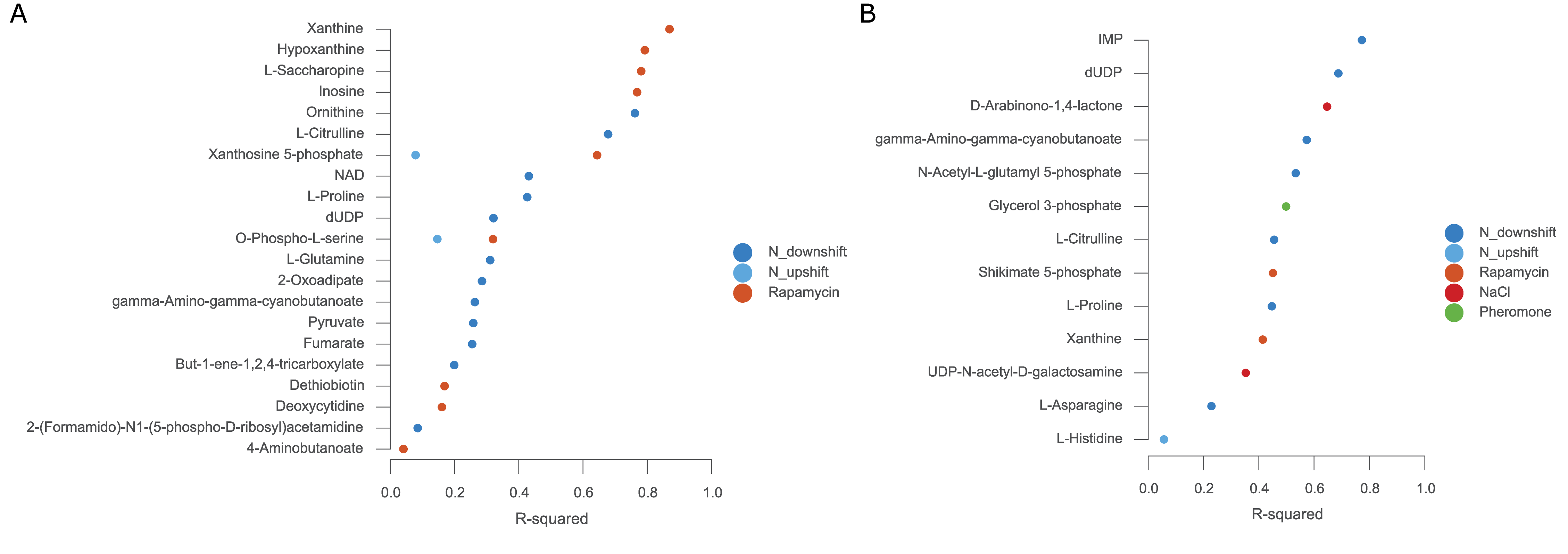

Supplement: S3 Fig — List of metabolites that displayed a positive coefficient of determination and significant Pearson correlation between the measured and predicted fold-changes across the different conditions. (TIF) [file pcbi.1005297.s003.tif]

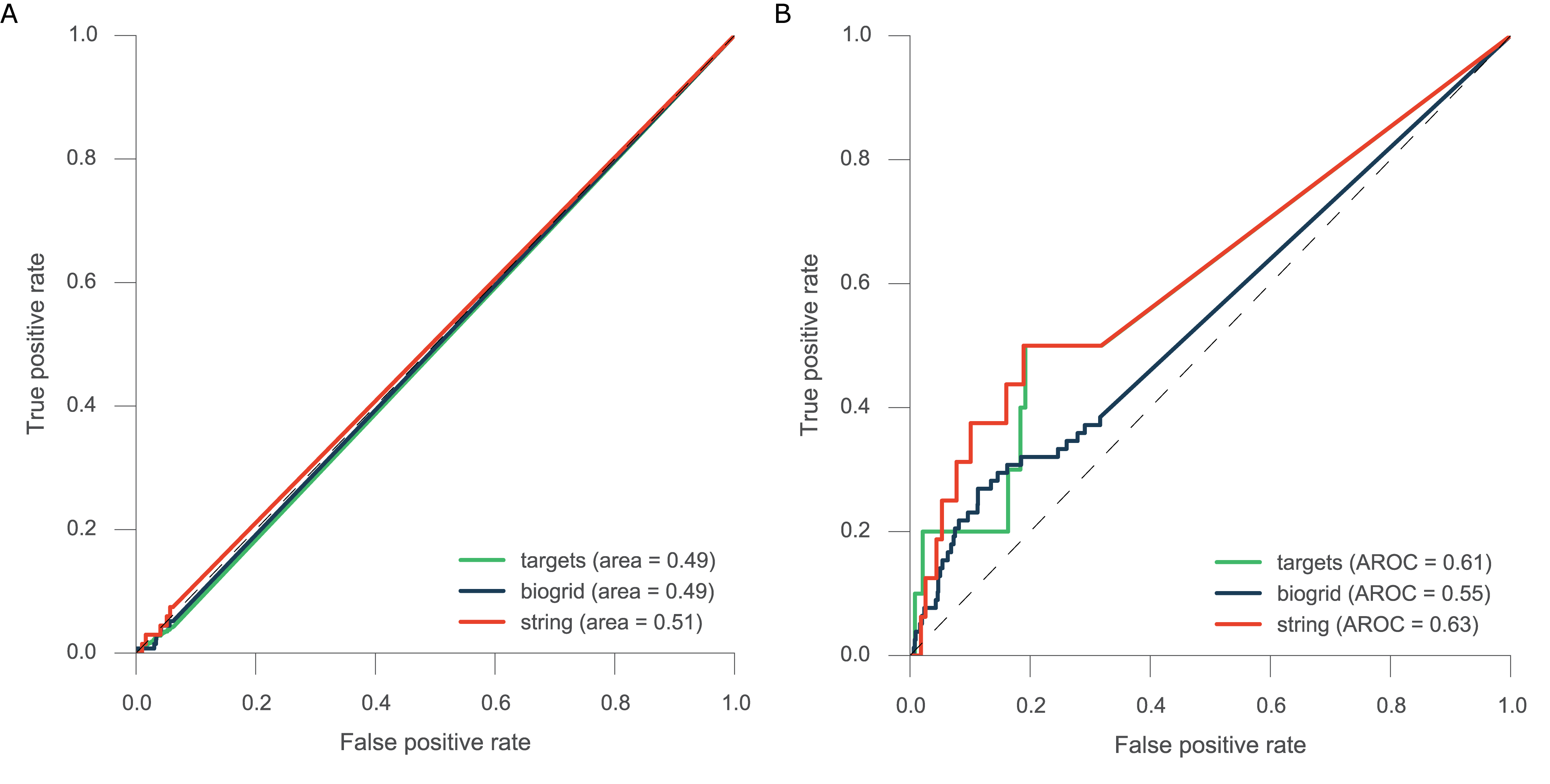

Supplement: S4 Fig — True-positive tables were built considering the specified resources. (TIF) [file pcbi.1005297.s004.tif]
